# Impact of the UN convention on the rights of persons with disabilities (UN-CRPD) on mental health care research - a systematic review

**DOI:** 10.1186/s12888-016-0862-1

**Published:** 2016-05-26

**Authors:** Christoph Steinert, Tilman Steinert, Erich Flammer, Susanne Jaeger

**Affiliations:** University of Mannheim, Chair for Political Science IV, Mannheim, Germany; Department of Psychiatry and Psychotherapy I, Centres for Psychiatry Suedwuerttemberg, Ulm University, Versorgungsforschung Weissenau, Ravensburg, Germany

**Keywords:** UN convention on the rights of persons with disabilities, Human rights, Inclusion, Mental health research

## Abstract

**Background:**

The United Nations Convention on the Rights of Persons with Disabilities (UN-CRPD) aims at stimulating profound changes and social development in many areas of the society. We wanted to examine the impact of the convention on mental health care research up to now by a systematic review.

**Methods:**

We searched relevant electronic databases for empirical studies from the area of mental health which focused directly on the content of the UN-CRPD.

**Results:**

One thousand six hundred ten articles were screened, 36 of which fulfilled the inclusion criteria and came from 22 different countries. 25 studies (69 %) are related to persons with intellectual disabilities, only 11 to other mental disorders. Study designs were quantitative and qualitative as well. Issues were realisation of the UN-CRPD, implementation and financing, development of instruments, and attitudes towards the UN-CRPD.

**Conclusions:**

In contrast to possible wide-reaching consequences for the organisation of mental health care, theoretical debates prevail as of yet and empirical research is still scarce. Research on the UN-CRPD is more advanced for intellectual disabilities and provides good suggestions for relevant research aspects in major mental disorders.

## Background

The United Nations (UN) Convention on the Rights of Persons with Disabilities (UN-CRPD) [1] was adopted by the United Nations General Assembly in 2006 and came into force in 2008. It was ratified by the European Union in 2010 and, in total, by 156 states in 2015. The UN-CRPD is the most recent in a row of UN human rights declarations, beginning from the Universal Declaration of Human Rights in 1948 [2]. The ratifying states acknowledge that the principles of the UN-CRPD should be transposed into their national legislations. Guiding principles are respect for inherent dignity, individual autonomy including the freedom to make one’s own choices, non-discrimination, full and effective participation and inclusion in society, equality of opportunity, and accessibility. The general idea of the UN-CRPD is inclusion of disabled people into the society and communities instead of establishing special rules and institutions [3]. Phrasing general principles of human rights, the UN-CRPD is completely applicable to the situation of people with mental illnesses as well as with other kinds of disabilities. Mental illness is not necessarily associated with disability; however, a clear distinction is not required since the UN-CRPD phrases principles which should be valid for all human beings, disabled or not. In the past two years, most interest has been attracted by article 12 (equal recognition before the law). This article does not contain any restrictions due to reduced mental capacity, which led to the idea that such a construct is generally not valid. Consequently in 2013, the special rapporteur on torture and other cruel, inhuman or degrading treatment or punishment of the UN Human Rights Council claimed to abolish all kind of coercive treatment as well as substituted decision-making legislations such as guardianship [4]. This has caused considerable concern among psychiatric organisations and stimulated ongoing controversies between psychiatrists and patients’ organisations in some countries [5]. However, this current and mostly critical discussion thwarts the engagement with the other aspects of the UN-CRPD – setting goals for mental health care that are not less challenging. The UN-CRPD aims at stimulating profound changes and social development in many areas of the society such as schools, workplace environment and public transportation. On this behalf, the convention calls for data gathering and research in the signatory states. Taking into account this and the huge political relevance of the UN-CRPD, we wanted to examine the impact of the convention on mental health research up to now by a systematic review.

## Methods

Our systematic review aims to identify studies that can be seen as a direct product of the impact of the UN-CRPD on mental health care research. For this purpose, a sequence of selection processes have been performed as described below.

The applied methods are in line with the Preferred Reporting Items for Systematic Reviews and Meta-Analyses (PRISMA) guidelines [6].

### Search strategy

Potentially relevant studies were identified by searching the electronic databases All EBM Reviews, Embase, Ovid MEDLINE(R), Ovid MEDLINE(R) In-Process & Other Non-Indexed Citations, PsycINFO and PSYNDEX plus Literature and Audiovisual Media. The searching process was performed in Ovid and conducted between the 3^rd^ of July and the 17^th^ of July 2015. We used the broadest possible search string consisting only of the nouns in the title of the UN-CRPD. Thus, we used the following search term in the multi-field search in Ovid: “convention” AND “rights” AND “persons” AND “disabilities”. Taking into account the possible national or regional character of research in this field, no language restrictions were made. This yielded 1610 articles.

### Inclusion criteria

Studies were selected in a two-stage process based on predefined inclusion and exclusion criteria.

In the first step, three criteria were applied. The first inclusion criterion was the requirement that a study was published in the context of the UN-CRPD. Consequently, we excluded articles which did not contain the term UN-CRPD (abbreviated or written-out) in its title, abstract or keywords.

The second inclusion criterion was the condition that the article could be classified as focused on the area of “mental health”. For the purpose of our study we defined mental health as mentioning of the terms “mental health“, “mental illness”, “mental disorder” or explicit mentioning of a diagnosis out of the International Statistical Classification of Diseases and Related Health Problems, 10th revision (ICD-10), chapter F (mental and behavioural disorders). Terms related to intellectual disability (ID, ICD-10 F 7x) such as “intellectual disability” and “mental retardation” also justified inclusion.

The third inclusion criterion required that a study could be classified as empirical. We defined empirical studies as all studies which are the product of research based on experimentation or observation. Thus, all studies were excluded which did not apply any form of qualitative or quantitative research. We assigned those excluded studies to three categories termed ‘legal discussion’ , ‘theoretical framework’ and ‘reviews or overviews’. The category ‘legal discussion’ refers to all studies which can be assigned to the juridical realm and which are based on norm-driven assumptions. ‘Theoretical framework’ delineates those articles which are based on theoretical concepts and their discussion. This category also includes case study-approaches without generalisations about a larger population. Finally, the category ‘reviews or overviews’ captures all non-systematic literature reviews, historical overviews, book reviews and general overviews about specific subject areas. All those excluded studies have in common that they did not produce or analyse real-world data.

**Fig. 1:**
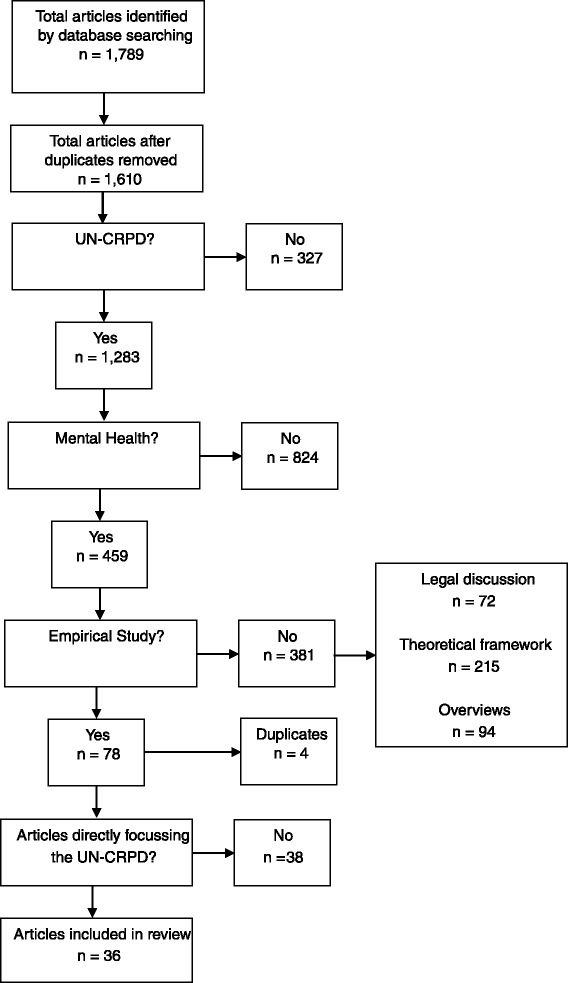
Flow diagram

We applied no a priori language restrictions to our search since we were confident to find competent translators in our multi-national hospital and research staff for all common languages. The Consort diagram of the selection process is displayed in Fig. 1. Deliberately, the first evaluation was rather lenient to avoid type II errors (false negative) at the expense of type I errors (false positive). The assessment of studies in the first-stage process was based on titles and abstracts.

### Study selection

In the second-stage process we identified those of the studies kept after the first-stage process that focused directly on the content of the UN-CRPD. Three authors (CS, SJ, EF) evaluated their full text content independently. An article was qualified as focusing on the UN-CRPD based on its research question or aim. To be included, the research question or aim must have targeted directly on the UN-CPRD. This condition was fulfilled when the research question either explicitly addressed the UN-CRPD or when it dealt with one of its general principles codified in Article 3. For the latter case, the concepts of inclusion and participation were central keywords.

Ambiguous cases were only included after discussions between the researchers. This final screening phase resulted in studies which were judged as directly inspired by the UN-CRPD. Those studies can be seen as direct product of the convention and their research was carried out in the light of the UN-CRPD.

## Results

In the first-stage process, we identified 1,610 studies. 327 studies were excluded because they did not cover the UN-CRPD, 824 studies were excluded because they did not cover mental health, and 381 studies were excluded because they were non-empirical. So after the first screening process 78 studies remained. After 4 duplicates had been removed, 74 articles were assessed on basis of the full-text content. In this second-stage process, 38 studies turned out to be not directly focussing on the content of the UN-CRPD. Eventually 36 articles could be included.

Those 36 relevant studies were categorized into two groups. The first group incorporates all publications in the area of intellectual disabilities (ICD-10 F 7x) which are displayed in Table 1. In total, there are 25 publications in the realm of intellectual disabilities representing 69 % of all included studies. The second group contains all publications on other mental disorders which are displayed in Table 2. There are 11 publications on mental disorders representing 31 % of the included studies. The included studies were carried out in 22 countries illustrating the global dimension of the UN-CRPD. The country with the highest number of studies is Australia (8, 22 %). The included studies employed multi-method approaches ranging from randomised controlled trials (RCTs) to different quantitative and qualitative approaches. In total, there are 2 RCTs (6 %), 17 quantitative studies (47 %) and 17 qualitative articles (47 %).

**Table 1:** Publications on intellectual disabilities

Relevant publications	Subject	Main results	Country	Materials and methods
Feldman et al. (2012) [31]	preliminary evaluation of health rights training	training group made significantly more correct responses on post training and follow-up tests	Canada	RCT with 6 month follow-up (*N* = 31)
Brolan et al. (2012) [32]	meaning, perceptions and experiences of advocacy by family members and paid support workers of adults with ID	advocacy roles are vital to people with ID	Australia	RCT (113 parents, 84 support workers of adults with ID)
Sermier Dessemontet & Bless (2013) [12]	the impact of including children with ID in general education classrooms with support on the academic achievement of their low-, average-, and high-achieving peers without disabilities	no significant difference in the progress of the low-, average- and high-achieving pupils from classrooms with or without inclusion	Switzerland	quasi-experimental study (*N* = 404)
Gray et al. (2014) [33]	changes in living arrangements and participation in daytime activities over time in a community population of young people with ID	adequate provision of accommodation and employment services for young adults with ID is lacking	Australia	quantitative longitudinal study (*N* = 536)
McConkey & Leavey (2013) [22]	changes from 2001 to 2011 in Irish attitudes towards the right to sexual fulfilment of persons with ID	in 2011, half the people in the survey thought that people with ID had the right to sexual relationships	Ireland	quantitative study in 2001 (*N* = 1000), in 2006 (*N* = 1004), in 2011 (*N* = 1039)
Badia et al. (2013) [16]	leisure activities of persons with ID	leisure activities and recreation activities were mostly solitary and passive in nature; age, type of schooling and severity of disability determine participation	Spain	cross-sectional quantitative study (*N* = 237)
Gobrial (2012) [24]	awareness of human rights of children with ID in Egypt	widespread lack of awareness of the rights of children with ID; respondents believed that these children had limited access to mental health care, social care, education and rehabilitation	Egypt	quantitative study, parents of children with ID (*N* = 72), professionals (*n* = 50), neither parents nor professionals (*n* = 78)
Stancliffe et al. (2011) [18]	benchmark on the degree of choice exercised by adult service users with ID in the USA	individuals living in their own home or an agency-operated apartment were more likely to choose where and with whom they live than individuals in nursing homes, institutions or group homes	USA	quantitative study (*N* = 6778)
Dusseljee et al. (2011) [34]	variations in community participation in the domains work, social contacts and leisure activities among people with ID	most people with ID in the Netherlands have work or other daytime activities, have social contacts and have leisure activities; people with ID in general hardly participate in activities with people without ID	Netherlands	quantitative study (*N* = 653)
Badia et al. (2011) [17]	influence of personal characteristics and environmental factors on the participation in leisure activities of people with ID	participation in leisure activities is determined more by personal factors and perceived barriers than by disability-related factors	Spain	cross-sectional quantitative study (*N* = 237)
Aznar et al. (2012) [26]	testing the usefulness of the ITINERIS scale on the rights of persons with intellectual disabilities (ISRPID)	the ISRPID can be an appropriate scale to monitor the UN-CRPD rights at an individual or group level	Chile	705 persons with ID, control group of 524 college students
Martin & Cobigo (2011) [35]	improving the understanding of the concept of social inclusion and its indicators	a clear definition of inclusion and its measurement is needed for decision-makers and service providers	Canada	retrospective analyses with adults residing in institutions (*N* = 1014) and with adults receiving community-based residential services (*n* = 327)
Drew et al. (2011) [7]	types of human rights violations experienced by people with mental and psychosocial disabilities in low-income and middle-income countries	wide range of human rights violations including the inability to access adequate mental health services or being subjected to stigma and discrimination	Belize, Bosnia and Herzegovina (and others)	survey (*N* = 51 people with mental and psychosocial disabilities from 18 countries) and review of literature
Fasching (2012) [36]	access to labour market measures to enhance vocational participation for people with ID	vocational guidance predominates qualifying measures and measures directly aiming at integration on the regular labour market	Austria	nationwide survey (*N* = 625 persons with ID participating in vocational measures)
Gomez et al. (2011) [37]	Exploratory investigation about implementation of human rights according to UN-CRPD	many situations of abuse and negligence are still existing. Violation of privacy recognized as major problem by both groups	Spain	quantitative study (*N* = 586 persons with ID in defined services and *N* = 161 professionals in the same services)
Garcia Iriarte et al. (2014) [38]	main issues for people with ID in Ireland	core concerns: Living options, employment, relationships, citizenship, leisure time, money management, self-advocacy and communication	Ireland	national study involving 23 focus groups (*N* = 168)
McConkey et al. (2013) [14]	inclusion within the context of Youth Unified Sports (which combines of players with ID and those without ID in the same sport teams) as perceived by athletes, partners, coaches, family carers and community representatives	factors which facilitate social inclusion of athletes: personal development of athletes and partners, creation of inclusive and equal bonds, promotion of positive perception of athletes, building alliances within local communities	Serbia, Poland, Ukraine, Germany, Hungary	qualitative study, 75 interviews in five different countries
O’Connor et al. (2012) [11]	lecturer responses to the inclusion of students with ID auditing undergraduate classes	the initiative was strongly supported by all lecturers, providing opportunity to consider more inclusive instructional approaches for all learners	Ireland	qualitative study (*N* = 11)
Saaltink et al. (2012) [39]	the right to participation for young people with ID in a family context	young people with ID follow an age-typical yet restricted pattern of participation in decisions about their lives; supported decision-making strategies are recommended	Canada	qualitative study (*N* = 10)
Hillman et al. (2012) [40]	issues related to human rights arising within the daily lives of people in personal support networks that included adults with ID	maintenance of rights within a supportive environment can be facilitated by deep knowledge, respect, promotion of active participation and provision of support	Australia	qualitative study, ethnographic study of 9 personal support networks
Shaw et al. (2011) [19]	views of people with ID and their family members regarding preferred models of housing and support for ageing people with ID	the main preference were models of housing that provide the opportunity to live in close proximity to their peers and in large groups in the community	Australia	qualitative study, focus group and individual interviews, adults with ID (*N* = 15) and family members (*n* = 10)
Kelly et al. (2009) [41]	views and experiences of Irish people with ID in the area of sexuality and relationships	people with ID are getting insufficient sex education	Ireland	qualitative study, focus groups (*N* = 15)
O’Brien et al. (2009) [10]	experiences of students with ID gaining access into a university setting	inclusion within the university setting led the students to see themselves more alike than different to their peers, they felt more accepted, more competent and more socially networked	Ireland	qualitative study, focus groups (*N* = 19)
Frawley & Bigby (2011) [13]	political orientations of advisory body members with ID, their participatory experiences and the types of support they received	the political perspective of members with ID varied; work was found hard but rewarding; both practical and intangible obstacles to participation were encountered	Australia	qualitative study, members of disability advisory bodies with ID (*N* = 9) and without ID (*n* = 12)
Cobigo (2014) [42]	lived experiences of persons with intellectual and developmental disabilities (IDD), identifying core components of the fundamental right of choice	four components identified: availability of choice opportunities, provision of choice options, informed cognitive process and act of choosing, supportive environment.	Canada	scoping review

**Table 2:** Publications on mental disorders

Relevant publications	Subject	Main results	Country	Materials and methods
Vijayalakshmi et al. (2013) [9]	the role of education in ascertaining human rights needs of people with mental illness	education is a mechanism for the pursuit of other human rights; empowerment to pursue education will play an important role in fulfilling the obligations of the UN-CRPD	India	quantitative study (*N* = 100)
Angermeyer et al. (2014) [23]	changes of public attitudes towards restrictions on mentally ill people	people’s views on patient rights have become more liberal, but the public is more inclined to restrict patients’ freedom in case of deviant behaviour	Germany	quantitative study, two population surveys (*N* = 2094; *n* = 3642)
Burns (2010) [43]	budget allocations over a 5-year period between psychiatric and general hospitals in KwaZulu-Natal	mean increase in budgets was considerably lower in psychiatric (3.8 %) than in general hospitals (10.2 %)	South Africa	quantitative study based on budget allocations (5 psychiatric and 7 general hospitals)
Steinert et al. (2015) [44]	Patterns of individual mobility and active use of motorised vehicles	Participants drove considerably less in time and distances than general population. Alcohol abuse and recurrent psychiatric hospitalisation were associated with exclusion	Germany	quantitative study (*N* = 150) with participants with schizophrenia or schizoaffective disorder
Kogstad (2009) [8]	violations of dignity considered from a clients’ point of view	gap between human rights’ aims and clients’ experiences in several settings; lack of safeguards against infringement	Norway	qualitative content analysis of 335 client narratives
Nomidou (2013) [25]	human rights in in-patient care in Greek mental health facilities using the WHO QualityRights toolkit	either improvement or initiation is necessary for the psychiatric clinic under research to fully comply with the requirements of the UN-CRPD	Greece	qualitative study, 21 in-depth interviews, documentation review and observation
Nankivell et al. (2013) [15]	orientation of nurses to human rights and access of consumers with severe mental illnesses to general practitioner services	the studied nurses only rarely raised the topic of human rights	Australia	qualitative study, 6 focus groups (*N* = 38)
Battams & Henderson (2012) [20]	current and potential impact of the UN-CRPD on Australian legislation and policy	there is a greater focus on concerns about ‘negative rights’ rather than ‘positive rights’; high rates of involuntary detention and a lack of access to the law for people with psychiatric disabilities continue to be significant problems	Australia	qualitative study, ten interviews with professionals from law, psychiatry, policy and service user backgrounds
Kleintjes et al. (2010) [21]	current support for mental health care user participation in policy development and implementation in South Africa	mental health care user consultation in policy development and implementation has been limited; however, most respondents felt that inclusion of user perspectives in policy processes would improve policy development	South Africa	qualitative study, semi-structured interviews (*N* = 96) and policy document analysis
Randall et al. (2012) [27]	producing a toolkit to document violations and good practice with the aim of preventing human rights violations and improving general health care practice in psychiatric and and social care institutions	the toolkit has demonstrated applicability and is qualified as acceptable and feasible for the systematic monitoring of human rights in psychiatric and social care institutions	UK (and others)	methodological and implementation study conducted across 15 European countries in monitoring visits to 87 mental health organizations
Henderson & Battams (2011) [45]	access and barriers to physical and mental health care	main barriers to the achievement to the right of health are structural (e.g. competing laws, political barriers)	Australia	qualitative study, interviews with 10 key stakeholders

Table 3 depicts a typology of all included studies from Tables 1 and 2. To categorise the multitude of diverse studies, we identified four major themes of research in light of the UN-CRPD. The first major theme is the realisation of the UN-CRPD and comprises all studies which investigate the gap between the aspirations of the convention and reality. While realisation consequently describes the practical fulfilment of the UN-CRPD, the second major theme termed ‘implementation and financing’ describes the legal fulfilment of the UN-CRPD. Studies belonging to this major theme analyse the implementation of the UN-CRPD in national legislation and as one specific aspect the financial framework as an indispensable condition for an effective implementation. The third major theme of studies inspired by the UN-CRPD is the development of instruments to capture the realisation of the convention. While the aim of those instruments is eventually to quantify the realisation of the UN-CRPD, articles belonging to this category focus rather on the construction of instruments than on the realisation as such. The last major theme we identified were attitudes towards the fulfilment of rights which are enshrined in the convention and the general awareness of the population of those rights. Studies belonging to this group analyse the realisation of the convention in people’s minds as an essential precondition for a realisation in practical terms. Taken together, there are 27 studies (75 %) focusing on the realisation of the UN-CRPD, 3 studies (8 %) investigating the implementation and financing, 3 articles (8 %) concerned with the development of instruments and 3 studies (8 %) analysing attitudes towards the UN-CRPD. Table 3 shows that 16 studies (44 %) apply a broad perspective focusing on the UN-CRPD as a whole, while 20 studies (56 %) investigate individual articles of the convention.

**Table 3:** Typology of publications in the context of the UN-CRPD

Major theme	Subthemes	Subject	References of relevant publications
Realisation	Key concerns of people with ID	UN-CRPD as a whole	[37, 38]
	Types of human rights violations	UN-CRPD as a whole	[7, 8]
	Living arrangements and participation in daytime activities of people with ID	UN-CRPD as a whole	[33]
	Impact of inclusion on peers without disabilities	UN-CRPD as a whole	[12]
	The right of participation of people with ID	UN-CRPD as a whole	[34, 39]
	Protection of rights in support networks	UN-CRPD as a whole	[40]
	Freedom of choice of persons with IDD	Article 3, 19 *(Choice)*	[42]
	Choice of living arrangements	Article 3, 19 *(Choice)*	[18, 19]
	Social inclusion of people with ID	Article 19 *(Social inclusion)*	[14, 35]
	Role of education in meeting human rights needs of people with mental illness	Article 24 *(Education)*	[9]
	Inclusion of people with ID in postsecondary education	Article 24 *(Education)*	[10, 11]
	Access to physical health care for people with serious mental illness	Article 9, 25 *(Health care services)*	[15, 45]
	Health advocacy	Article 25 *(Health)*	[31, 32]
	Inclusion in political and public life	Article 29 *(Participation in political and public life)*	[13]
	Patterns of leisure participation of people with developmental disabilities	Article 30 *(Leisure participation)*	[16, 17]
	The right to sexual self-determination	Article 23, 25 *(Sexual self-determination)*	[41]
	Access to labour market for people with ID	Article 27 *(Work and employment)*	[36]
	Use of motorized vehicles	Article 20 *(personal mobility)*	[44]
Implementation, Financing	Budget allocations to psychiatric hospitals	UN-CRPD as a whole	[43]
	Impact of UN-CRPD on national legislation and policy	UN-CRPD as a whole	[20]
	Mental health care user participation in policy development and implementation	Article 29 *(Participation in political and public life)*	[21]
Development of instruments	Application of ISRPID to measure the extent to which people with ID exercise their rights	UN-CRPD as a whole	[26]
	Development of ITHACA toolkit for systematic monitoring of human rights violations	UN-CRPD as a whole	[27]
	Application of the WHO QualityRights toolkit which is based on the UN-CRPD	UN-CRPD as a whole	[25]
Attitudes	Public attitudes towards restrictions on mentally ill people	UN-CRPD as a whole	[23]
	Awareness of human rights of children with ID	UN-CRPD as a whole	[24]
	Public attitudes towards the right of sexual fulfilment of people with ID	Article 23, 25 *(Sexual self-determination)*	[22]

## Discussion

The most important result of this review was what it did not find: an appropriate amount of research in mental health care reflecting the importance of the UN-CRPD. Empirical research on aspects of the UN-CRPD is still scarce. This is in sharp contrast with the huge impact it could and probably will have on the complete organisation and structure of mental health care in all ratifying countries. 25 out of 36 studies that could be included (69 %) were related to intellectual disabilities, which was an unexpected result. Intellectual disabilities fall into the definition of mental disorders as classified in the ICD-10, chapter F (F 7) and thus fulfilled our inclusion criteria, but in most countries service provision and care is separate from other mental disorders. In published articles, the term ‘mental disorder’ typically refers to psychotic and affective disorders, though intellectual disabilities and dementia are also classified as mental disorders. None of the included articles referred to dementia or acquired brain injury. Anyway, the detected body of research on intellectual disabilities provides valuable insight on important research topics which should be put on the agenda for other severe mental disorders.

The majority of studies inspired by the convention analyses to what extent the aspirations of the convention are realised in a real-world context. Drew et al. [7] find a wide range of violations of the convention’s aims in low-income and middle-income countries. Kogstad [8] demonstrates that there is also a gap between human rights aims and patients’ experiences in a high-income country. To bridge the gap between aspirations and reality, Vijayalakshmi et al. [9] assign the right to education a catalyst function. Successful examples of realisation of the UN-CRPD are represented by the studies by O’Brien et al. [10] and O’Connor et al. [11] who show the positive effects of inclusion in postsecondary education. In the same vein, Sermier Dessemontet and Bless [12] demonstrate that there is no negative impact of inclusion on the achievements of non-disabled peers. Furthermore, the studies of Frawley and Bigby [13] and McConkey et al. [14] represent best-practice examples for the fulfilment of the principles of the convention. Other studies focus on the realisation of individual aspects of the convention such as access to physical care [15], participation in leisure activities [16, 17] or the choice of living arrangements [18, 19]*.* While those studies offer valuable insights, there is still a lack of systematic studies investigating the realisation of the UN-CRPD as a whole.

Moreover, there is a paucity of research about the implementation of the convention into national legislation. Battams and Henderson [20] provide such a study for the context of Australia and Kleintjes et al. [21] analyse the specific aspect of mental health care user participation in policy development and implementation. While there is a multitude of articles in the judicial realm analysing and discussing the implementation of the UN-CRPD, there is a lack of empirical studies concerned with this question. Also studies investigating attitudes towards the rights in the UN-CRPD and capturing the awareness of those rights are still rare. McConkey and Leavey [22] provide such a study specifically for Irish attitudes towards the right to sexual fulfilment of persons with intellectual disability. Angermeyer et al. [23] have a broader scope investigating general changes in public attitudes towards restrictions on mentally ill people. Gobrial’s [24] study analyses the public awareness of children’s rights with intellectual disability in Egypt. While those studies are interesting on their own, they just offer insights on specific attitudes about particular rights. There is lack of studies capturing the general awareness of the convention and general attitudes towards its meaning in the population. Finally, there are some remarkable attempts to develop instruments to capture the realisation of the UN-CRPD. While Nomidou’s [25] study is based on the World Health Organization (WHO) QualityRights toolkit, Aznar et al. [26] have developed a particular instrument called ISRPID to capture the realisation of the rights of persons with intellectual disabilities. Similarly, Randall et al. [27] have developed the Institutional Treatment, Human Rights and Care Assessment (ITHACA) toolkit which allows a systematic monitoring of the rights enshrined in the UN-CRPD. Those instruments have a huge potential to serve as a base for further studies investigating the realisation of the convention’s rights.

Our review has several limitations. First of all, we may have applied too narrow inclusion criteria that limited the scope of possibly relevant studies. For instance, no example of peer research, i.e. participatory research by mental health service users, could be included. The few research papers we could find had a focus slightly different from issues in the UN-CRPD, e.g. they focused on everyday experiences and individual coping strategies [28, 29]. Although they are covering important topics that are related to rights of people with disabilities the research was not explicitly ‘a direct product of the UN-CRPD’ as was required in this review paper. This is indeed a shortcoming of our inclusion criteria. Similarly, we could not include some methodological articles on the improvement of methods for the research in people with particular disabilities (e.g. specific interview techniques, participatory research) although they addressed a necessary precondition of the research we were interested in [30]. Further, the reliability of the criteria could be questioned. To rate studies as empirical studies in the area of mental health and in the context of the UN-CRPD seems quite straightforward. But whether a study focused directly on the content of the UN-CRPD is to some degree vague. It is possible that other researchers would have come to a somewhat different set of included studies.

## Conclusions

There is urgent need for further research in all areas covered by the UN-CRPD relating to mental disorders. Much more evidence is needed for answering the many questions that are posed by the UN-CRPD, such as how inclusion and equality can be realised for people disabled by mental disorders and what impact a change of policies, service provision, legislation, and attitudes would have on their mental health. Up to now, the different research projects have been rather isolated and often focused on specific subject areas. While the UN-CRPD has inspired several studies, those studies are rather disconnected projects than part of a coherent body of research. More research has been done in the field of intellectual disabilities as compared to the field of severe mental disorders. This offers interesting suggestions to learn from. We therefore recommend systematic and ideally transnational research projects investigating the realisation and implementation of the UN-CRPD. In particular, in the subfield of mental disorders there is still a paucity of research about the achievements of the UN-CRPD. Further research in this area would contribute to pave the way for an effective realisation of the basic principles enshrined in the UN-CRPD.

## Abbreviations

ICD-10: International Statistical Classification of Diseases and Related Health Problems, 10th revision
ID: intellectual disabilities
IDD: intellectual and developmental disabilities
ISRPID: ITINERIS scale on the rights of persons with intellectual disabilities
ITHACA: Institutional Treatment, Human Rights and Care Assessment
PRISMA: Preferred Reporting Items for Systematic Reviews and Meta-Analyses
RCT: randomised controlled trial
UK: United Kingdom
UN: United Nations
UN-CRPD: United Nations Convention on the Rights of Persons with Disabilities
USA: United States of America
WHO: World Health Organization
